# Cognitive Health and Differential Cortical Functioning in Dissociative Trance: An Explorative Study About Mediumship

**DOI:** 10.3389/fpsyg.2022.874720

**Published:** 2022-04-11

**Authors:** Karleth Costa Spindola-Rodrigues, Renandro de Carvalho Reis, Caio Macedo de Carvalho, Socorro D’Paula Nayh Leite Loiola de Siqueira, Antonio Vitor da Rocha Neto, Kelson James Almeida

**Affiliations:** ^1^Pharmaceutical Sciences Post-graduation Program, Federal University of Piauí, Teresina, Brazil; ^2^Department of Neurology, Federal University of Piauí, Teresina, Brazil

**Keywords:** mediumship, cognitive function, cognitive health, Brazil, dissociative phenomenon

## Abstract

**Aim:**

To evaluate the cognitive functioning of subjects practicing trance mediumship in Brazil.

**Method:**

The study was based on the measurement of cognitive functions of 19 spirits mediums through neuropsychological tests such as the Brief Cognitive Screening Battery (BCSB), the Verbal Fluency Test (FAS), the digit span test, the cube test, the five digit test (FDT) and an evaluation of mental health through scales such as the Beck Depression Inventory (BDI), the Self-Report Questionnaire (SRQ), and the Trauma History Questionnaire (THQ). The sample included the participation of spirit mediums divided into two groups. The more experienced group (MEG) with 11 subjects had more than 10 years of mediumistic practice, while the other less experienced group (LEG) with 8 subjects had 1–5 years of experience. The inclusion criteria were psychophonic mediums (who have the ability to communication when deceased beings communicate directly via speaking) with regular trance practices for at least one year. The data collected were analyzed using the SPSS statistical package.

**Results:**

Regarding performance on the BCSB and digit span test, all subjects reached scores at the median or higher in comparison to standardized scores of Brazilians. Scores of 90% on the cube test and 42% on the FAS were reached in comparison to median or higher values, versus the median of standardized scores among Brazilians. On the FDT, we found statistical significance (*p* = 0.038) in the choice stage, with higher performance of subjects whose initial age of trance recognition occurred before 21 years old. On the BDI scale, no participant met the criteria for major depression. The SRQ showed an incidence of common mental disorders in 21% of the sample, which was more prevalent in the LEG (*p* = 0.008).

**Conclusion:**

The cognitive functioning of subjects who practice trance mediumship in Brazil is associated with cognitive health. Executive dysfunction may be a tendency in LEG. However, an incidence of common mental disorders in the LEG was observed. Executive processing was higher in the subgroup with early practices of recognizing the phenomenon.

## Introduction

*Mediumship* is a spiritual phenomenon displayed in different cultures and religious traditions; it has been reported throughout the history of humanity, where a person claims to communicate with deceased people (Moreira-Almeida et al., 2008). In this situation, there are reports that associate this phenomenon with the total and/or partial loss of consciousness (Bastos et al., 2018) and the ability to make judgments. Rituals in various forms are regularly performed in both religious and non-religious contexts, and numerous cultures around the world believe that this form of communication provides truthful information (Wahbeh et al., 2019).

Mediumistic experiences are usually dissociative, such as motor automatisms that involve writing, sensory, cognitive language, or more complex situations such as in “possession” (Peres et al., 2012). These are understudied cultural phenomena. Although nonpathological dissociation is quite common in the general population, dissociative experiences are studied as a risk factor for dissociative disorder or pathology (Facco et al., 2019). The DSM-5 states that such dissociative experiences should not be viewed as inherently pathological (American Psychiatric Association, 2013). In some studies, essential differences between dissociative disorder and mediumship were 221 verified, in which mediumship was considered to be a nonpathological form of dissociation (Mainieri et al., 2017; Vencio et al., 2019) and a protective factor against mental health conditions (Moreira-Almeida et al., 2008). In this research, Moreira-Almeida et al. studied 115 Brazilian mediums and showed adequate mental health in this group.

Analysis of cognitive performance, associated with dissociative experiences, is scarce in the literature. There is only one publication that has demonstrated neuropsychological findings, focusing on the evaluation of cognitive functions in people with altered states of consciousness (Al-Adawi et al., 2019). This study revealed executive dysfunction in so-called “spirit possession” among practitioners of only one type of trance mediumship.

Dissociation is commonly understood as a kind of built-in defense mechanism that allows individuals to psychologically protect themselves from extreme emotions and excitation triggered by a traumatic event (Vinhosa Bastos et al., 2018).

Thus, diagnosed executive dysfunction may result from a higher prevalence of traumatic experiences, from the difference between trance and possession regarding the preservation of frontal circuits, or from the quality of thought content during trance. Hence, there is a need to analyze the cognitive performance of subjects in other populations with different subtypes of trance beyond possession. The main research question is if mediumistic trance practice may impair the cognitive process. Our research hypothesis is that practitioners of mediumistic trance have preserved cognitive performance.

Therefore, we aimed to evaluate cognitive health among practitioners of mediumistic trance. In addition, cognitive performance and cortical functioning can be evaluated by focusing age of the person when the experience occurred, mental health comorbidities (such as previous traumatic experiences), and early recognition of mediumship according to milestones for neuronal specification.

## Methods

Nineteen—19 subjects completed all steps of this research; they were native Brazilians without residence abroad who regularly participate in religious activities of Kardecist Spiritism. Spiritism is a faith of which approximately 3 million Brazilians are reported to be followers (Instituto Brasileiro de Geografia e Estatística, 2010), and has intrinsic cultural components in which some of its members report having dissociative, mediumistic trance experiences. Research authorization was granted by the local Research Ethics Committee (#3.922.991). The study complied with the Declaration of Helsinki, with Brazilian legislation of resolutions 466/12 and 510/16 regarding research involving human beings.

### Study Setting and Participants

The research was carried out in cities in the northeastern region of Brazil. The recruitment of participants was performed using the snowball sampling strategy, a technique in which a participant indicates another who meets the necessary requirement to participate. This is a non-probability sampling method that can be useful when the researcher is not able to known the number of persons with this ability in the population. The study staff did not known the participants identify during sociodemographic questionnaire applying. They only received their identity in order to complete the cognitive testing.

Nineteen psychophonic mediums who regularly attended Spiritist centers were included. These subjects reported having the ability to communication when deceased “beings” communicate directly via speaking. After initial selection, the subjects were divided into two groups. The first was considered to be the more “experienced” group (MEG) with 11 subjects, while the second was considered to be the less “experienced” group (LEG) with 8 subjects. Experience was defined by the length of time they had practiced mediumship (weekly or monthly). For the MEG, more than 10 years and a minimum of 1 trance activity per week were taken into account. For the LEG, 1–5 years of practice and up to 4 practices of the phenomenon per month were accepted. The period between 6 and 9 years was considered intermediate (the intermediate group).

Subjects who were absent from the activities developed in the Spiritist centers for more than 1 year were excluded, as well as those with decompensated psychiatric disorders (depression, schizophrenia), reports of sleep disorders (such as parasomnias, REM sleep behavioral disorder), a diagnosis of epilepsy according to the criteria of the International League Against Epilepsy (ILAE), subjects on psychoactive medications, reports of drug use or abuse, and subjects in the intermediate group.

A comparison of neuropsychological performance between the MEG and LEG was performed to analyze the differences in cortical processing. In previous studies with neuroimaging in meditation using the mindfulness technique, different patterns of cortical activity and higher speed in executive processing have been observed depending on the years that subjects had previously practiced meditation (Falcone and Jerram, 2018).

### Procedures

The data from the sociodemographic questionnaire were collected using Google forms; the participant’s identity and any form of identification were concealed. The following data were requested: date of birth, gender, level of education, profession/occupation, ethnicity, and marital status.

For the purposes of inclusive criteria, they were asked about the ability to enter into a mediumistic trance, especially in laboratory and scientific research environments; the period when they started practicing mediumship; what type of mediumship they practiced (psychophonic, psychographic, clairvoyant); how often they entered into a mediumistic trance, weekly and/or monthly; and how the participant characterized their state of consciousness during mediumistic communication (conscious, semiconscious, unconscious).

To verify the exclusion criteria, they were asked about the existence of previous diseases; if they continuously and/or daily used psychiatric medications; if they had ever had a seizure or sleep disorder; and if the time of mediumistic practice corresponded to the intermediate period of 6–9 years.

Due to the COVID-19 pandemic, we sought to preserve the participants’ health by maintaining face-to-face contact only for the application of neuropsychological tests. Thus, some instruments were adapted to an online format via Google forms: The informed consent form, the Beck Depression Inventory (BDI), the Self-Report Questionnaire (SRQ), and the Trauma History Questionnaire (THQ) were used exclusively for research purposes. After verifying the inclusion criteria, neuropsychological tests were applied, and cognitive functions were evaluated.

#### Cognitive Assessment

All tests and scales used have been validated and/or studied for use in the Brazilian population, so the scores obtained were compared with the values from standardized tables and their respective cutoff points, as well as stratification of the subjects according to expected scores for age and education. A rationale for choosing the cognitive and mental health tests/questionnaire was the use of tests that allowed analyzing each cognitive domain and diagnosing cognitive impairment accurately.

The **Brief Cognitive Screening Battery** (BCSB) is a battery of tests for the evaluation of perception/identification, naming, incidental memory, immediate memory, and late memory (Nitrini et al., 2004). To evaluate perception and naming, the participant was presented with a sheet of paper containing 10 images and asked to name each image. The numbers of items evoked provided the late memory score (1–4: below average; 5: average; 6–10 above average).

**The verbal fluency test (FAS)**, considered “interference” in the previous battery, was applied. The FAS test was used to request the production of words obeying the initial letter rule. In addition to assessing components of executive function, it can measure the ability of controlled oral word association. The total score was given by summing all correct words beginning with the three letters (F, A, S); for statistical analysis, the score obtained was converted into percentiles (5–25: below average; 50: average; 75–95: above average) (Marquine et al., 2021).

The BDI is a self-assessment scale consisting of 21 groups of statements where depressive characteristics/attitudes and symptoms are quantified. Each category describes a specific behavioral manifestation of depression (Beck et al., 1961). The diagnosis of major depression will be categorized with a BDI > 19 points.

The **SRQ** is a self-report questionnaire (Harding et al., 1980) validated for the Portuguese language (Mari and Williams, 1986). It consists of 20 questions designed to identify common mental disorders at the primary care level, which are characterized by non-psychotic symptoms such as insomnia, fatigue, irritability, forgetfulness, difficulty concentrating, and somatic complaints (Oliveira Bernardes Santos et al., 1970). Eight or more positive answers (yes) suggest the presence common mental disorders.

The THQ is a self-report questionnaire adapted to Portuguese (Fiszman et al., 2005), composed of 24 questions with “yes” or “no” answers divided into three fields: 4 questions related to traumatic situations involving crimes, 13 questions related to trauma in general and disasters, and 6 questions related to the experience of physical and/or sexual violence. For the purpose of this statistical calculation, we used the number of “yes” answers.

The **digit span test** is one of 15 subtests of the *Wechsler Adult Intelligence Scale* (WAIS), which was developed as a measure of attention, concentration, and memory (Glassmire et al., 2016). On this test, progressive sequences of numerals were presented orally, whereby the examinee reproduced them immediately after the presentation. The test is applied in two stages. In the first or direct stage, the examinee repeats the sequence of digits in the same order as presented. In the second, or indirect, stage, the digits are reproduced in the opposite order to the one presented by the examiner. The score considered for statistical analysis will be the weighted score (0–19) obtained from the absolute number of digits repeated (0–6: low performance; 7–12: average; 13–19: above average).

The **Wechsler Abbreviated Scale of Intelligence** (WASI) is a brief, reliable measure of intelligence in clinical and research contexts. It can be applied to people aged 6–89, and provides scores for verbal IQ, performance IQ, and total IQ. It is composed of four subtests: vocabulary, cubes, similarities, and matrix reasoning. The cubes subtest was chosen because it assesses skills related to spatial visualization, visuomotor coordination, and abstract conceptualization (i.e., it gauges perceptual organization and general intelligence) (Hilsabeck et al., 2003). On this subtest, the participant uses the cubes to reproduce 13 two-color models within a given time period, which progress in increasing difficulty, starting with two cubes, the simplest, up to nine cubes, the most complex. The score considered for statistical analysis will be the score (0–6: low performance; 7–12: average; 13–19: above average) obtained from the absolute number of digits repeated.

The **five digit test** (FDT) evaluates an individual’s speed and mental efficiency in any language. It is a test of cognitive functions based on simple linguistic concepts: reading digits from 1 to 5, counting elements from 1 to 5, and producing words using the quantities “one,” “two,” “three,” “four,” and “five.” The FDT has four stages associated with different cognitive processes that can be grouped into automatic (reading and counting) and controlled (inhibition and choice) processes; these provide information about certain mental processes: (1) the overall speed of cognitive processing; (2) verbal fluency (i.e., the ability to find the words one wants to say); (3) the participant’s focused attention and his/her reaction to continued effort; (4) the participant’s ability to muster the additional cognitive effort needed to control involuntary responses and switch between two different mental operations. The score considered for statistical analysis will be the score obtained for reading, counting, inhibition, choice, flexibility, and inhibition in percentiles (5, 25, 50, 75, 95). To categorize them, 5–25 percentiles are below average, 50 on the average and 75–95 above average.

## Data Organization and Statistical Analysis

The data collected were tabulated in Microsoft Windows Excel and analyzed with the help of the SPSS statistical software package version 20.0 (IBM Corporation, Armonk, United States). Windows Excel tables were used to characterize the participants’ profiles and the sociodemographic questionnaire.

The performances in neuropsychological tests were categorized as below or above the mean according to previously validated means to the Brazilian population. These performances were defined as dependent variables. The independent variables were socio-demographic variables (group type, gender, age, schooling time, and marital status). A ROC curve defined age and schooling time categories after visual choices looking for the best areas under the curve. The chi-square test and its complementary tests (Fisher or Likelihood ratio test) evaluated the difference between the MEG and LEG and the relevance of the results collected on the neuropsychological tests. Differences were considered significant at p < 0.05.

## Results

### Sample Characteristics

The participants had a mean age of 45.1 ± 9.8. The mean age at which the manifestation of mediumship was perceived was 21.4 ± 12.9 years. The sample was 68% female and 32% male.

There were 8 individuals in the LEG, representing 42% of the sample, and 11 in the MEG, denoting 58%. Regarding the level of consciousness during the mediumistic trance state, 58% were conscious, 32% were semiconscious, and 11% were unconscious. Those who reported being unconscious belonged to the MEG group.

As for level of education, 37% of the participants had up to 12 years of schooling, and 63% had 12 or more years of schooling. Regarding marital status, 37% were in a stable relationship, and 63% lived alone or with relatives.

### Psychiatric Findings

The SRQ showed an incidence of common mental disorders in 21% of the sample (*p* = 0.008, chi-square test), all belonging to the LEG, according to Table 1.

**TABLE 1:** Frequencies of participants showing common mental disorders by Self Report Psychiatric Screening Questionnaire (SRQ) with respect to the group and the socio-demographic variables in subjects practicing trance mediumship.

Variables	*n*	SRQ		
		≥8	<8	*p*
Group type				0.008
LEG	8	4	0	
MEG	11	0	15	
Gender				0.75
Female	13	3	10	
Male	6	1	5	
Age*				0.906
<21	10	2	8	
≥21	9	2	7	
Schooling time (years)				0.188
≤12 years	7	1	6	
> 12 years	12	3	9	
Marital status				0.539
Cohabits with partner %.	7	2	5	
Lives alone or with relatives	12	2	10	

*Early age of perception of mediumship.

Using the BDI, major depression was ruled out in 100% of the subjects. On the THQ, 100% of the sample mentioned having experienced at least one traumatic event, 10% had only experienced trauma related to crime events, 26% had only experienced trauma in general and during disasters, 31% had experienced situations related to crime and trauma in general, and 31% had experienced trauma related to physical and/or sexual violence. Those who experienced physical/sexual violence had also experienced all other events, as shown in Table 2.

**TABLE 2:** Performances in Trauma History Questionnaire (THQ) and Beck Depression Inventory (BDI) scales with respect to group and socio-demographic variables in subjects practicing trance mediumship.

Variables	*n*	THQ						BDI	
		Crimes	*p*	General trauma	*p*	Physical and sexual	*p*	Depression	No depression
Group type			0.345		0.202		0.636		
LEG	8	0		8		2		0	8
MEG	11	2		9		4		0	11
Gender			0.637		0.310		0.911		
Female	13	0		11		4		0	13
Male	6	0		6		2		0	6
Age*			0.701		0.115		0.405		
<21	10	0		10		4		0	11
≥21	9	1		7		2		0	8
Schooling time (years)			0.210		0.289		0.677		
≤12 years	7	1		6		1		0	7
>12 years	12	0		11		5		0	12
Marital status			0.865		0.253		0.419		
Cohabits with partner %.	7	0		7		3		0	7
Lives alone or with relatives %	12	1		10		3		0	12

*Early age of perception of mediumship.

### Cognitive Performance

In the evaluation of cognitive functions, on the BCSB, 100% of the subjects exhibited scores in the average and/or above average range at the end of the test, which evaluates late memory. The same outcome was found on the digit span subtest of the WASI; 100% of the subjects performed above or equal to the average found among Brazilian individuals. On the cubes subtest, 90% of the subjects scored higher than or equal to the average. On the FAS (verbal fluency) test, only 42% of the subjects scored higher than or equal to the Brazilian average (31% in the MEG and 10% in the LEG), as seen in Table 3.

**TABLE 3:** Performances in FAS, CUBES, DIGITS subtest with respect to group and socio-demographic variables in subjects practicing trance mediumship.

Variables	*n*	FAS			CUBES			DIGITS			BCSB—Late M.		
		<mean	≥mean	*p*	<mean	≥mean	*p*	<mean	≥mean	*p*	<mean	≥mean	*p*
Group type				0.198			0.763			0.348			0.228
LEG	8	6	2		1	7		0	8		0	8	
MEG	11	5	6		1	10		0	11		0	11	
Gender				0.127			0.412			0.943			0.13
Female	13	6	7		2	11		0	13		0	13	
Male	6	5	1		0	6		0	6		0	6	
Age*				0.845			0.277			0.596			0.33
<21	10	6	4		0	10		0	10		0	10	
≥21	9	5	4		2	7		0	7		0	9	
Schooling time (years)				0.598			0.36			0.198			0.556
≤12 years	7	5	2		1	6		0	7		0	7	
>12 years	12	6	6		1	11		0	12		0	12	
Marital status				0.361			0.405			0.891			0.179
Cohabits with partner %	7	5	2		0	7		0	7		0	7	
Lives alone or with relatives %	12	6	6		2	10		0	12		0	12	

*Early age of perception of mediumship.

On the FDT test, in the evaluation of the automatic processes that involve reading and counting, 53% showed percentiles equal to or higher than the average, of which 64% belonged to the MEG. In the controlled choice process, 68% had percentiles equal to or higher than the average; of these subjects, 64% belonged to the MEG. When the FDT subtest named controlled choice process was performed, there was statistical significance related to the age of perception of mediumship, with *p* = 0.038—chi-square test (Table 4).

**TABLE 4:** Performances in five digit test (FDT) test with respect to group and socio-demographic variables in subjects practicing trance mediumship.

Variables	*n*	FDT																	
		AP. reading			AP. counting			CP. choice			CP. alternance			Inibition			Flexibility		
		<mean	≥mean	*p*	<mean	≥mean	*p*	<mean	≥mean	*p*	<mean	≥mean	*P*	<mean	≥mean	*p*	<mean	≥mean	*p*
Group type				0.432			0.307			0.763			0.095			0.425			0.507
LEG	8	5	3		5	3		2	6		1	7		2	6		1	7	
MEG	11	4	7		4	7		4	7		6	5		6	5		4	7	
Gender				0.667			0.5			0.988			0.895			0.664			0.634
Female	13	7	6		7	6		4	9		5	8		6	7		3	10	
Male	6	2	4		2	4		2	4		2	4		2	4		2	4	
Age*				0.084			0.067			0.038			0.081			0.063			0.723
<21	10	6	4		7	2		1	9		2	8		2	8		2	8	
≥21	9	3	6		2	7		5	4		5	4		6	3		3	6	
Schooling time (years)				0.579			0.888			0.909			0.676			0.125			0.22
≤12 years	7	5	2		5	2		2	5		2	5		3	4		3	4	
>12 years	12	4	8		4	8		4	8		5	7		5	7		2	10	
Marital status				0.652			0.78			0.283			0.119			0.839			0.589
Stable relationship %	7	4	3		4	3		2	5		3	4		3	4		1	6	
Lives alone or with relatives %	12	5	7		5	7		4	8		4	8		5	7		4	8	

*Early age of perception of mediumship.

Regarding performance in controlled process alternation, 63% had a score higher than or equal to the average; of these subjects, 45% belonged to the MEG. In the evaluation of inhibitory control, 58% had a score higher than or equal to the average, and 45% belonged to the MEG. On the cognitive function of flexibility, 74% had a score higher than or equal to the mean, and 64% belonged to the MEG.

## Discussion

The present study performed a broad neuropsychological evaluation of subjects who regularly participate in cultural or religious activities in which they experience dissociative mediumistic trance phenomena. The results were able to suggest cognitive health regarding the different cognitive domains evaluated: executive functioning, memory, visuospatial functioning, attention, and language. In addition, an exploratory analysis was performed that included comparisons between subjects with regard to descriptive epidemiological variables of the sample, and the same analysis was stratified into subgroups according to the time of recognition and perceptions of mediumship.

Worse performance was observed in brief psychiatric assessment scores in the LEG. The depression scores of both groups validated the findings in the following neuropsychological evaluation. Both groups performed above the average related to the population regarding the tests assessing visuospatial functioning and memory. Concerning the tests that evaluated frontal processing, there was a tendency for the MEG to perform better than the general population average in the following modalities: cognitive flexibility, the automatic processes of reading and counting, and the controlled process of choice. Subjects who experienced dissociative mediumistic trance phenomena before the age of 21 exhibited above-average frontal cortical processing in the choice control scores, which was statistically significant.

### Mental Health in Dissociative Mediumistic Trance

In a comparative study between mediums and individuals with dissociative identity disorder, mediums differed from persons with dissociative identity disorder, indicating better social adjustment, lower prevalence of mental disorders, lower use of mental health services, no use of antipsychotics, a lower prevalence of histories of physical or sexual abuse in childhood, and sleepwalking (Moreira-Almeida et al., 2008). As a result, mediumship stood out, demonstrating better mental health and social adjustment. Other research compared spiritualist mental mediums to non-medium spiritualists. It suggested that mediums had better psychological wellbeing and reported lower psychological distress (Roxburgh and Roe, 2011). Another study with 3,023 participants suggested that individuals who claimed mediumship had higher dissociation scores than non-claimants, but neither group exceeded the threshold for pathology (Wahbeh and Radin, 2017).

According to Bastos et al. (2018), during mediums’ training, prior to communicating with dead individuals, one of the most important skills to be acquired is control over the manifestations of possession. There is a role of learning this control of the possession phenomenon, which focuses on reading and education, as to the principles of the spiritism and behavioral adjustment to cultural rules (Espirito Santo, 2010). This process reflects broad acquisition and application of aspects of executive processing, with inhibitory control over the possession phenomenon, as well as cognitive flexibility for changes in relation to the trance state and readjustment to the real environment.

Spirit possession cannot be considered pathological in relation to mental health, except when it causes clinically significant discomfort and impairment in social and occupational functioning (van Duijl et al., 2010). However, in this study, a difference was found in the prevalence of common mental disorders only in the LEG (i.e., in the group with less time of regular study and systematic activities aimed at controlling mediumship). It is inferred that the regularity of practices in the cultural and religious aspects of this practice may favor the concept of spirit possession as a phenomenon and not a disorder.

Another study suggested that performance on the verbal fluency test was significantly different among participants with intermittent dissociative phenomena when compared to transient dissociative phenomena (Al-Adawi et al., 2019). Such a finding did not corroborate the current study’s outcomes. Although 58% of the participants scored below average on the FAS test, there was no statistical significance between the groups. However, a below-average performance only on the FAS test—which was not observed in the digit span test and in all FDT subtests—makes executive dysfunction unlikely, even when stratifying the groups into MEG and LEG. It is hypothesized that the cognitive impairment observed in the study by Al-Adawi et al. (2019) may be related to the thought content experienced during possession phenomena.

Brain areas associated with the preservation and learning of memory, attention, and executive functioning tend to be dysfunctional in people with dissociative phenomena (Cima et al., 2001). Dissociation can be characterized by subtle deficits in neuropsychological performance, such as attention impairment, and some cognitive phenomena associated with dissociation seem to be dependent on the emotional or attentional context (Giesbrecht et al., 2008). However, in general, the mediums in this study exhibited scores within the Brazilian average on neuropsychological tests for the assessment of long-term memory, working memory, visuo-constructive ability, and planning. It is necessary to differentiate dissociative disorder from dissociative phenomena related to cultural experiences. Cognitive health also seems to follow this trend.

The fast, efficient reproduction of a series of elements on the FDT test indicates not only the presence of focused attention, but also the ability for automatization, learning, resistance to fatigue, and inhibitory control (Broverman et al., 1966). In the choice process, several variables are verified at the same time: attention, phonology, semantics, control, and inhibition of one response and activation of another. In our study, there was statistical significance between the age the medium began to exhibit mediumistic abilities and their scores within the choice process (a lower age of onset was associated with a higher score in this FDT subtest). We are unaware of studies that address such a comparison. There is probably a greater cortical susceptibility to the dissociative mediumistic trance phenomenon in some subjects, or the learning process and cortical specialization are facilitated by the early onset of mediumistic recognition. The phenomenon of neuronal pruning occurs until the age of 21, and is fundamental in the specialization of the functionality of individuals and preparation for the cognitive challenges that the individual will come across (Holguera and Desplan, 2018).

Mediumistic trance can be understood as a form of mediation in which an individual voluntarily access degrees of states of conscious. Both strategies (meditation and trance) may allow the subjects to access their ego-conscious (Wahbeh et al., 2019). Some studies showed an improvement in cortical activity by meditation using the mindfulness technique (Tanaka et al., 2014; Taren et al., 2017; Wang et al., 2020). Functional MRI neuroimaging studies have shown a change in the pattern of cortical activation between less experienced and more experienced mediumship practitioners. While human insula activation is observed in less experienced meditation practitioners, more experienced meditation practitioners activate areas of the right medial frontal gyrus, anterior cingulate gyrus, globus pallidus, and putamen (Falcone and Jerram, 2018).

In Brazil, the study conducted by Peres et al. (2012) with psychographic mediums (communication with deceased beings through writing) showed that among more experienced mediums, who wrote while in the trance state, cerebral blood flow was consistently lower in the regions responsible for complex writing compared to the control condition. During the mediumistic trance, there was low activity in the brain areas responsible for complex writing in the MEG. In this PET study, the more experienced mediums reported deeper trance states in contrast to the less experienced mediums. Cortical neuroimaging changes can be further detailed by thorough neuropsychological evaluation. In the present study, there was a tendency for better performance on tests that assessed subgroups of frontal tasks, suggesting better executive processing associated with the practice of dissociative, mediumistic trance phenomena.

## Strengths and Limitations

This study has strong points: Provides insight that may help understand cognitive skills in practitioners of mediumistic trance. Contradicts previous findings from Al-Adawi et al. (2019) because an executive dysfunction was highly prevalent in practitioners of possession, and seeks to support future studies to define the risk of cognitive impairment in practitioners of mediumistic trance.

This study has limitations related to its design. As cross-sectional research, we may only describe frequencies and associations. A study with a prospective design may suggest an independent risk of cognitive impairment in practitioners of mediumistic trance. We presented a small sample of subjects because a broad neuropsychological assessment is challenging in volunteers. Small sample sizes usually have low statistical power, less precise estimations of the population parameters, and inaccurate generalization inferences regarding the reference population. Further studies could analyze the quality of emotions and thought content associated with this dissociative phenomenon. It may explore the depth of the mediumistic trance and the level of consciousness during it, comparing different groups looking for differences beyond trance and possession. Functional neuroimaging studies with cognitive tasks aimed at executive functioning may clarify some limitations.

## Conclusion

Religiosity and its relationship with the unknown have always aroused interest, curiosity, fear, and distrust in many people. Thus, science has been developing ways to prove and/or question what has traditionally been believed through faith (Moreira-Almeida et al., 2008; Tanaka et al., 2014; Taren et al., 2017; Wang et al., 2020).

Through the analysis of religiosity, cultural manifestations can interfere with cortical functioning and cognitive health. The present study revealed that the cognitive functioning of Brazilian mediums, whether with much or little experience in activities related to this cultural experience, was equal to or above the average value considered normal for the Brazilian population.

In general, there was cognitive health among Brazilians who practiced trance mediumship as a cultural experience. However, we observed a higher prevalence of psychiatric impairment in the LEG, and executive dysfunction may be a tendency in LEG. Performance in processing in the frontal cortex was better in the subgroup with early onset of recognition of the mediumistic phenomenon. Diagnosed executive dysfunction may result from a higher prevalence of traumatic experiences, the impairment of frontal circuits on LEG, or the quality of thought content during trance.

## Data Availability Statement

The raw data supporting the conclusions of this article will be made available by the authors, without undue reservation.

## Ethics Statement

The studies involving human participants were reviewed and approved by Facid Research Ethics Committee #3.922.991. The patients/participants provided their written informed consent to participate in this study.

## Author Contributions

KA and KS-R: conceptualization, methodology, resources, and writing—review and editing. RR: data curation and formal analysis. KS-R, CC, AR, and SS: investigation and writing—original draft. KA: project administration and supervision. KA, KS-R, and RR: validation and visualization. All authors contributed to the article and approved the submitted version.

## Conflict of Interest

The authors declare that the research was conducted in the absence of any commercial or financial relationships that could be construed as a potential conflict of interest.

## Publisher’s Note

All claims expressed in this article are solely those of the authors and do not necessarily represent those of their affiliated organizations, or those of the publisher, the editors and the reviewers. Any product that may be evaluated in this article, or claim that may be made by its manufacturer, is not guaranteed or endorsed by the publisher.
